# Supplementary material for: Exploring stroke survivors’ and physiotherapists’ perspectives of the potential for markerless motion capture technology in community rehabilitation
Source: J Neuroeng Rehabil. 2024 Sep 20;21:168. doi: 10.1186/s12984-024-01467-x (PMC11414257; doi:10.1186/s12984-024-01467-x)
Supplement: Supplementary file 2 — Supplementary Material 2 [file 12984_2024_1467_MOESM2_ESM.docx]

# Stroke rehabilitation system specifications

## Essential

The proposed technology must assess and monitor the exercises being carried out by a stroke survivor and share that information back with the therapist such that they can adapt the exercises to the needs of the user. The following features were suggested as essential for the technology to be beneficial to both stroke survivors and therapists.

**System User and user environment**

Assistive devices - Stroke survivors often have assistive devices which they may well be wearing or using while carrying out their exercises. The technology must be able to function with a user who is wearing or using an assistive device which may affect the way that they move e.g. AFO, zimmer frame. Patients may not have assistive devices but may need to use a rigid body for support when carrying out exercises e.g. Otago exercises.

Exercise equipment – Some exercises may involve the stroke survivor interacting with items e.g. weights, kettlebells, elastic bands, Therabands etc. The technology should be able to cope with these additional items.

Exercise position – Post-stroke rehabilitation exercises can be carried out in a range of positions e.g. sitting/standing/lying down. The software will need to be able to cater for these positions.

Exercise environments – Stroke survivors and therapists both described conducting exercises in multiple rooms around the house, often next to furniture or in positions with little space around e.g. in the kitchen next to the countertop, on the bottom steps of the staircase. The technology will need to be able to move around the house as needed for the user – could it potentially function from behind the patient if space was limited and behind had the clearest view of the movements?

Patient may not be the only person in the frame – The software will need to consider situations where: The therapist is in the camera view helping the patient with their exercises (can this be done?), and where a partner or person is sitting in the room with the patient, either in a position where they are watching or supporting with exercises, or if they are engaging with different activities in the background.

Patient needs to be able to see themselves while exercising and system needs to be movable – Given that exercises are carried out across the house, the system will need to be able to be easy to move and adjust to focus on parts of the body as needed. Having a way for patients to be able to see themselves while exercising is very important, whether this through projection of the software onto a TV screen or through paired devices.

**User feedback**

Markers of progress – Technology will need to have clear measures of failure or progress that can be quickly and easily monitored and understood by users.

Performance metrics - As part of rehabilitation, exercises are likely to stay the same but the performance metrics may change. The red/amber/green categorisation may not always be useful for stroke rehab and an alternative may need to be considered, we will need to develop metrics to show improvement or worsening for this system, these should be sensitive to fatigue.

Session length – The system will need to be able to consider the amount of time that the patient spends exercising in the day. It will need to monitor fatigue and notice when the user’s performance is deteriorating. If performance is deteriorating it needs to end the session, if performance is not deteriorating then the system needs to encourage activity. Too much/too little deterioration could act as a marker for progression/reassessment by therapist.

**Exercises**

It needs to be very clear what the technology can not be used for:
• e.g. what exercises can this technology not incorporate?
• Can it be used to train a person to get up from the floor?
• Can it be used to identify if someone has fallen during an exercise?
• Will all exercises be preinstalled?
• Will the exercises be able to focus on specific-limbs and wider body parts as needed?

Solo or Supported exercises marked – There is a risk around getting patients to reach the boundaries of what they can do in therapy at home. The technology needs to be able to consider whether patient will be by themselves and whether it could be unsafe for them to carry out exercises alone. Participants suggested that they would like the exercises to be marked so that they knew whether the activity was safe for them to do alone or whether it would be safer to carry them out with a carer around. It would be nice to be able to select a regime based on whether the stroke survivor is alone or accompanied.

**Accessibility**

Software needs to be accessible to all users – Stroke survivor ability is extremely diverse. The software needs to be able to be used by individuals with a range of physical and cognitive abilities. Patients will need to be able to move tech themselves and set it up - so it has to be easy to understand where to put it/move it etc.

User friendly –System needs to be very user friendly and easy to engage with. Any data or charts that it produces need to be easy for the user to understand quickly and without any professional interpretation.

Audio feedback – Some stroke survivors have visual problems associated with their stroke. Stroke survivors and therapists both suggested that an audio function built into the software that could guide them through the process or give some verbal feedback would be beneficial. It may be appropriate for this guidance or feedback to be very direct, with minimal words.

**Service**

Service – To benefit the stroke survivors, this technology will need to be underpinned by a service which provides and maintains the therapeutic elements of the rehabilitation. i.e. it will need to be sold to and through a professional gatekeeper.

Therapist – Stroke survivors were keen to feel that the technology was supported by a trained professional or therapist. Though participants suggested that this individual wouldn’t necessarily have to be a physiotherapist but could be another trained health professional.

Professional needs to be integrated into the service – Both stroke survivors and therapists were keen that the technology should be accompanied with checkups with a medical professional overseeing their rehabilitation, whether in person or using video conference software. Wanting therapy with a person is not always about the technology, the visits from the therapist provide a social link for people who may not see many other people. Regular checkins with a therapist or a function where users could exercise with another stroke survivor or a trained peer mentor (e.g. from a Stroke club or the Stroke Association) could increase usage.

Supported tuition pre-discharge – Technology would need to be incorporated prior to discharge from hospital so that the stroke survivor has an opportunity to learn how to use it, ask any questions, and is reassured at the start that there is a therapist monitoring them at the other end of the technology.

Progression – It would be nice to have a clear measure of progression over time so that the user can easily monitor their development. Is there a way of comparing baselines across weeks of exercises in the reports?

**Logistics**

Cost is likely to be an issue – Both therapists and stroke survivors discussed cost as a limiting factor to engage with this technology, particularly the cost of the equipment to host the software. Therapists suggested that they would be interested in a subscription-based package E.g. 10 licenses a month that you can allocated them to those who need them most. They indicated that obtaining funding is likely to be hard and said that they will need significant evidence-based research to present a strong case to their commissioners.

Concerns about how the tech will integrate with existing systems – To be useful for therapists, the technology will need to be able to link into existing documenting systems so that the data can be shared with other staff e.g. System One, though this may vary across Trusts. We will need an API to interface the metrics from this system to hospital based electronic records or GP records.

Concerns about data collection – The technology will be in private homes, therefore it needs to be very secure and only controlled by the patient.

**Other**

Reassurance – Stroke survivors were concerned that the technology would be able to give the same level of support and feedback/guidance as a person. The software should aim to reassure them, where possible, that a therapist is involved with the administration of the exercises and is monitoring them, that the software itself is sensitive enough to observe their movements and measure them accurately, and that the technology will provide specific help if they need it.

User characteristics – Technology must be able to work accurately with patients of any body size, skin colour, with or without facial or body hair. Users will also exercise in a range of clothes, if there are limitations with the system for some exercises regarding loose clothing, this could be expressed in the software as a pop-up comment or similar which provides guidance about what clothing to wear.

## Desirable

The following features were suggested as desirable for the software.

**Software**

Motivational elements – Many stroke survivors suggested that some type of motivational element would be beneficial for them e.g peer support, games, targets etc. Others suggested this would be a drawback for them, so if motivational elements were included, an option to opt in/out would be nice. Stroke survivors described how as time progresses after a stroke, it can become harder to self-motivate to engage with the exercises. Something to support this in the early stage and establish exercise as a habit would be beneficial.

**Service**

Safety - Would be nice for the technology to incorporate a recognition if someone has fallen over/ask if they’re okay (perhaps identify if user is OK via hand signals) and guide them up or send a message to someone to provide help.

Technology to incorporate general feedback from user – It would be nice to incorporate a series of scores or scales about the user: e.g. how they found the exercises and wider information about patient wellness e.g. any sickness, slept poorly, mental fatigue etc. Data on perceived exertion could be another way to notice progression – the user may not be doing more of the exercise than they were two weeks ago, but they may be finding it easier to do.

## Desirable future steps

**Involving further technology**

Thermal camera – it would be useful to have a mechanism that would monitor the temperature of users at the start of the exercise slot such that it could detect if they were unwell and issue a warning to not exercise until they are feeling better.

Integrate other Apple devices or data – Could the software incorporate an Apple watch? This would provide heart rate data and potentially also include the potential to monitor walks/step data and include them in the exercise data. Ability to walk places is often a goal for patients - so tech/therapy could be more meaningful to patients. This could well be a second phase of rehab – the user may have progressed past the iPad scheme and been discharged from their services, this could support them further in the community.

Move away from the Apple market as the technology progresses – Stroke survivors suggested that it would limit them if technology was only ever released through Apple and would like to be able to use the software on devices that they already have.

**Service**

The proposed technology could also be beneficial within existing services - The technology itself could be useful for use by therapists with clients who would be less suitable for personal use at home as currently proposed or within exercise groups within hospitals/clinics. The ability to see distribution of weight while doing exercises could be beneficial to therapists.
